# Fabrication and Tribological Properties of Epoxy Nanocomposites Reinforced by MoS_2_ Nanosheets and Aligned MWCNTs

**DOI:** 10.3390/ma17194745

**Published:** 2024-09-27

**Authors:** Zhe Tong, Jiaxuan Du, Xiangmeng Li, Zeyu Liu, Chao Yan, Wenxing Lei

**Affiliations:** 1 School of Mechanical Engineering, North University of China, Taiyuan 030051, China; 2 Shanxi Key Laboratory of Advanced Manufacturing Technology, Taiyuan 030051, China; 3 Shanxi Jiangyang Chemical, Ltd., Taiyuan 030041, China

**Keywords:** epoxy nanocomposites, electric field inducement, aligned MWCNTs, tribological properties

## Abstract

The epoxy nanocomposites reinforced by MoS_2_ nanosheets and aligned multi-walled carbon nanotubes (MWCNTs) were fabricated by DC electric field inducement. The epoxy nanocomposites achieved improvement in the tribological properties with the addition of randomly dispersed MoS_2_ and MWCNTs compared to the pure epoxy. Furthermore, the epoxy nanocomposites exhibit anisotropic tribological and mechanical properties when the MWCNTs are aligned in the composites. The tribological properties of epoxy nanocomposites containing 1 wt% MoS_2_ and aligned 1.2 wt% MWCNTs achieved the maximum improvement when the sliding direction is perpendicular to the axial direction of MWCNTs. Compared to random MoS_2_ nanosheets and random MWCNTs reinforced epoxy nanocomposites, the friction coefficient and wear rate of random MoS_2_ and aligned MWCNTs reinforced epoxy nanocomposites decreased by 11.3 and 66.7% under a load of 5 N, respectively. The increased thermal conductivity and mechanical properties, higher surface content of nanoparticles, as well as unique alignment mode of MWCNTs are considered to be the main reasons for the improvement of tribological properties of epoxy nanocomposites.

## 1. Introduction

Epoxy composites have attracted enormous research interests in many industrial fields, such as automobile, aerospace, etc., because of their low cost, excellent mechanical performance, and the advantage of being designable [1,2]. However, the insufficient hardness, toughness, and thermal conductivity have hindered the application of epoxy matrix composites as friction components. Various modified techniques thus have been developed to improve the performance of epoxy matrix nanocomposites [3,4,5].

CNTs and graphene have been widely deployed in many industrial fields due to their unique structure and excellent mechanical and thermal conductive properties [6,7,8]. The corrosion protection, mechanical properties, and tribological properties of epoxy matrix nanocomposites are enhanced by the addition of CNTs, and the enhancement mechanisms are related to the excellent interfacial combination between CNTs and matrix, as well as the prominent hardness of CNTs [9,10,11,12,13]. Furthermore, the modification of the crystal structure of metallic composite has been obtained by adding CNTs, and the carrying capacity and lubricating properties have thus been significantly improved [14]. To meet the increasing performance requirements, the modification technology of various fillers has been proposed, which takes advantage of the synergistic effect of different types of additives [15,16,17]. The lubricating property and wear resistance of polymer matrix composites can achieve effective improvement by virtue of the high mechanical properties of CNTs and low interfacial shear strength of 2D additives [18,19,20,21].

Several kinds of one-dimensional materials, especially CNTs, generally possess anisotropy in mechanical, thermal, and electrical conductivity properties due to their unique structure. Thus, the macroscopic physical properties of polymer nanocomposites reinforced by one-dimensional materials are highly dependent on the orientation of the one-dimensional materials [22]. The wear resistance of epoxy matrix composites containing continuously aligned carbon nanotubes (ACNTs) achieved significant improvement through the wetting grown arrays method. In particular, the wear rate reduced from 3727.2 to 0.83 × 10^−6^ mm^3^/N·m when sliding along the normal orientation of the CNTs [23]. The mechanical properties, electrical conductivity, and erosive wear resistance of epoxy composites can be more efficiently improved by filling aligned CNTs or graphene [24,25,26,27,28]. In addition to the CVD method, the CNTs or graphene can achieve alignment by applying electric field induction due to their dielectric properties and high length-to-diameter ratio or laminated structure. Meanwhile, electric field induction has the advantage of low cost and time consumption compared to the CVD method [29,30]. In addition, the CNTs show more effective alignment after subjected functionalization because of the increased dispersity of CNTs in the liquid medium [31,32]. The halloysite nanotubes (HNTs) have been aligned in epoxy using a nozzle spray coating method. The results showed that the mechanical performances and wear resistance of epoxy composites achieved marked improvement in the out-of-plane direction [33]. However, the addition of such one- or two-dimensional fillers in combination with other nanoparticles into polymer matrix composites to modify their tribological properties has not been previously reported.

In this study, with the aim of enhancing the tribological and mechanical properties of epoxy composites, the epoxy nanocomposites containing both MoS_2_ nanosheets and aligned MWCNTs were prepared by applying an external direct-current (DC) electric field. We investigated the mechanical and tribological properties of epoxy nanocomposites containing MoS_2_ nanosheets and aligned MWCNTs. The results indicated that the mechanical and tribological properties of epoxy nanocomposites containing MoS_2_ nanosheets and aligned MWCNTs were significantly improved compared to pure epoxy and that of containing random MWCNTs, especially in the normal orientation of MWCNTs due to the anisotropy of MWCNTs and the high-quality transfer film on the sliding counterpart’s surface.

## 2. Materials and Experiment

### 2.1. Materials

Sulfourea (CSN_2_H_4_) was purchased from Tianjin TIANLI Chemical Reagents Ltd., Tianjin, China, and ammonium molybdate ((NH_4_)_6_Mo_7_O_24_·4H_2_O) was purchased from Tianjin Chemical Reagent 4th Factory Kaida Chemical Plant, Tianjin, China. Silane coupling agent (KH 550) was provided by Huai’an He Yuan Chemical Co., Ltd., Huai’an, China. Concentrated sulfuric acid, concentrated nitric acid, oxalic acid, and the other chemical reagents are analytical grade. MWCNTs were purchased from Nanjing Xianfeng Nanomaterials Technology Co., Ltd., Nanjing, China. The epoxy resin (EPOLAM 2040) and hardener (EPOLAM 2042) were purchased from Sika-Axson Group, Shanghai, China. Concentrated sulfuric acid and concentrated nitric acid are analytical reagents. Distilled water was used during the experimental process.

### 2.2. Epoxy Nanocomposites Preparation

#### 2.2.1. Synthesis of MoS_2_ Nanosheets

The synthesis of MoS_2_ nanoparticles was conducted according to the procedures described by Bei Bei Chen et al. [34]. The typical preparation process was as follows: 1.24 g (NH_4_)_6_Mo_7_O_24_·4H_2_O and 2.28 g CSN_2_H_4_ were added to 36 mL distilled water with magnetic stirring for 15 min. Subsequently, the solution was poured into a Teflon-stainless steel autoclave (50 mL) and kept at 220 °C for 24 h. Then, the reaction product was collected via vacuum filtration when the temperature of the autoclave dropped down to room temperature and was then washed with distilled water several times. Finally, the product was dried at 75 °C in a vacuum oven for 24 h.

#### 2.2.2. Surface Treatment of MoS_2_ Nanosheets

The silane coupling agent (KH 550) was employed to enhance the dispersibility of MoS_2_ in epoxy resin by modifying its surface. The specific process was as follows: KH 550 (2 g) was added to a mixture (50 mL) of ethyl alcohol and distilled water (volume ratio of 9:1). Subsequently, an appropriate amount of oxalic acid was added to the mixture until the pH of the mixture was below 4.5. After stirring for 1 h, 40 mg of MoS_2_ nanosheets were added to the mixture, followed by stirring for 4 h under 80 °C. Finally, the resulting precipitate was collected via vacuum filtration, followed by washing with distilled water and ethyl alcohol three times. The final precipitate was dried under a vacuum environment at 60 °C.

#### 2.2.3. Functionalization of MWCNTs

MWCNTs (100 mg) were introduced into a mixture (40 g) (concentrated sulfuric acid was mixed with concentrated nitric acid with a weight ratio of 3:1), followed by stirring at 80 °C for 2 h. Subsequently, the MWCNTs were collected via vacuum filtration carefully and washed with distilled water several times until the pH of the filtrate reached neutral. Finally, the filter cake was dried under a vacuum environment for 24 h at 60 °C.

#### 2.2.4. Fabrication of Epoxy Nanocomposite Containing MoS_2_ Nanosheets and Aligned MWCNTs

**Figure 1:**
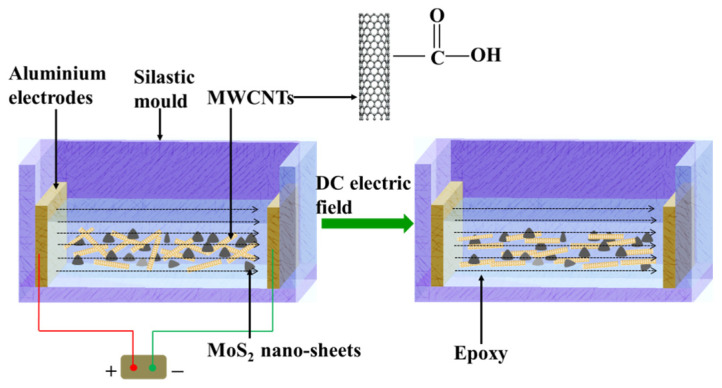
Schematic of the fabrication process of epoxy nanocomposites containing MoS_2_ nanosheets and aligned MWCNTs.

The epoxy nanocomposites containing MoS_2_ nanosheets and aligned MWCNTs were fabricated using the casting method with DC electric field inducement, as illustrated in Figure 1. In this study, the epoxy nanocomposites containing 1 wt% of MoS_2_ nanosheets and five different contents of MWCNTs (0.3, 0.6, 0.9, 1.2, and 1.5 wt%) were studied. In a typical fabrication process, the appropriate mass of MoS_2_ nanosheets and MWCNTs was dispersed in 10 mL of anhydrous alcohol by ultrasonic treatment for 0.5 h. Subsequently, the suspension was added to the epoxy (15 g) and stirred for 0.5 h, followed by ultrasonic treatment for 1 h. Subsequently, the mixture was transferred to a vacuum chamber for 6 h to remove the solvent. Next, a 5 g hardener was added to the mixture through stirring. Afterward, the mixture was poured into a silastic mold and degassed in vacuum conditions. Finally, the mixture was cured with the presence of a DC electric field (The electric field strength was 70 V_p-p_/mm at 5 kHz) under 30 °C for 10 h. The electric field remained constant throughout this curing process. Subsequently, the resin was cured further under 70 °C for 16 h. Two aluminum plates (50 × 15 × 3 mm^3^) were used as electrodes for applying the DC electric field.

The pure epoxy and epoxy nanocomposites containing random MoS_2_ nanosheets and MWCNTs were also fabricated according to the same procedures without the DC electric field induction. The fabricated epoxy nanocomposites containing random and aligned nanocomposites are denoted as R-composites and A-composites, respectively.

### 2.3. Characterizations

The microstructure characteristics of MWCNTs and MoS_2_ nanosheets were characterized using a field emission scanning electron microscope (SEM, Gemini SEM 500 at 10 kV, New York, NY, USA) and a transmission electron microscope (TEM Hitachi H-7650 at 80 Kv, Tokyo, Japan). The crystalline phase of synthesized MoS_2_ nanosheets was determined with an X-ray diffractometer (XRD D8 Advance A25 at 40 kV and 40 mA of Cu Kα, Bruker, Karlsruhe, Germany). The surface functional group of oxidized MWCNTs was characterized by a Fourier transform infrared spectroscopy (FTIR Nicolet iS50, Madison, WI, USA). The worn surface morphologies of epoxy nanocomposites were analyzed using SEM. X-ray photoelectron spectroscopy (XPS, Thermo Field, Waltham, MA, USA) was performed to analyze the elements and chemical composition of the worn surface of the composite, using C1s as the reference combining energy.

In order to observe the alignment of nanoparticles in composites, a 10 mL epoxy solution containing low concentration MWCNTs (0.05 wt%) and MoS_2_ nanosheet (0.05 wt%) was inducted by using a DC electric field. The optical microscope and digital camera were used to characterize the alignment of nanoparticles in the liquid epoxy. Moreover, the position state of MWCNTs in nanocomposites was characterized by observing the cross-section of nanocomposites by using SEM.

### 2.4. Theromechanical and Tribological Properties Tests

The compressive strength of epoxy nanocomposites was tested according to ASTM D695-23 with a tensile electromechanical universal testing machine (INSTRON-5982, INSTRON, Boston, MA, USA) [35]. The loading rate was 1.3 mm/min. The hardness of epoxy nanocomposites was measured with a Vickers hardness tester (BEIJING TIME TMVS-1, Beijing, China), and the indenter load was set as 0.98 N. The thermal conductivity of epoxy nanocomposites was tested by using a laser thermal conductivity meter (NETZSCH LFA 447 German, Selb, Germany) at room temperature and 150 °C.

The tribological behaviors of epoxy nanocomposites were evaluated using a UMT-2 multifunctional friction testing machine (UMT-2, CETR, Bethesda, MD, USA) under dry conditions at room temperature with a reciprocating length of 6 mm and a sliding frequency of 1 Hz. The stainless-steel ball (Φ 9.6 mm) with a surface roughness of approximately Ra = 0.22 μm was used as the sliding counterpart. The normal loads applied were 5 N, 10 N, and 15 N. For the A-composites, the sliding direction included two modes: one sliding direction was vertical, and the other was parallel to the MWCNTs’ axial direction. The corresponding friction test results are denoted as A-composites-V and A-composites-P, respectively. The wear volumes of epoxy nanocomposites were measured by using a laser scanning confocal microscope (Olympus OLS4000, Tokyo, Japan).

## 3. Results and Discussion

### 3.1. Formation of MoS_2_ Nanosheets and MWCNTs

**Figure 2:**
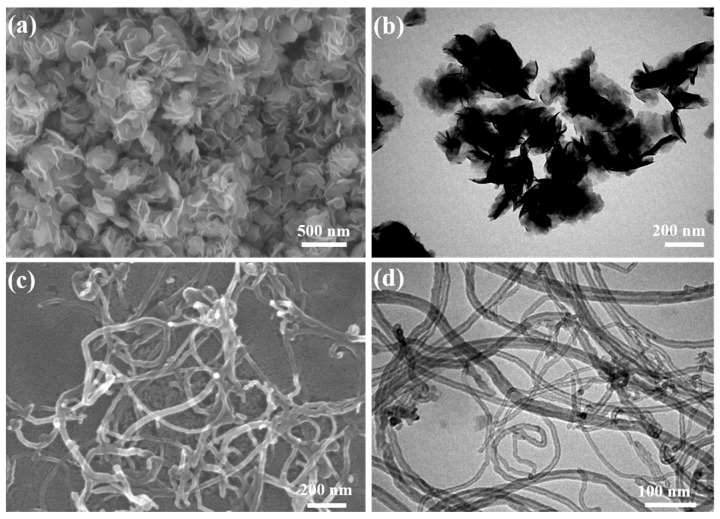
SEM (**a**) and TEM (**b**) micrographs of the MoS2 nanosheets; SEM (**c**) and TEM (**d**) of the modified MWCNTs.

The microstructure of MoS_2_ nanosheets and modified MWCNTs is shown in Figure 2. It is evident from Figure 2a that the MoS_2_ exhibits a sheet-like structure, with a diameter ranging from 300 to 500 nm and a thickness of 10–30 nm. Furthermore, Figure 2b reveals the presence of surface wrinkles on the sheets, indicating the multilayered nature. In Figure 2c,d, the MWCNTs exhibit diameters ranging from 20 to 50 nm, with an approximate length of 10 μm. Notably, even after modification treatment, the surface of the MWCNTs remains relatively intact.

**Figure 3:**
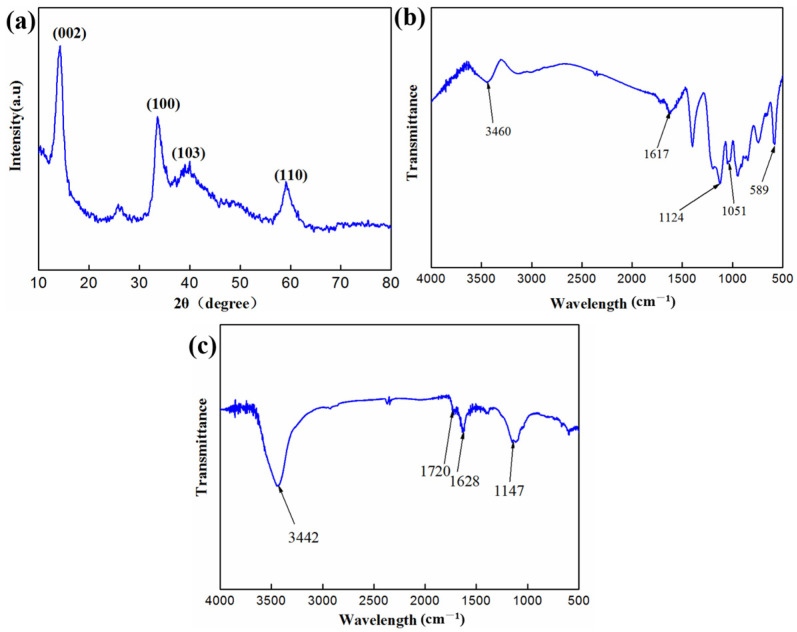
XRD curve (**a**) and FT-IR spectra (**b**) of MoS2 nanosheets treated with KH-550; (**c**) FT-IR spectra of oxidized MWCNTs.

Figure 3 shows the XRD curve and FT-IR spectra of MoS_2_ nanosheets and MWCNTs. In Figure 3a, the diffraction peaks 2*θ* at 14.4°, 32.9°, 39.3°, and 59.1° correspond to the (002), (100), (103), and (110) plane of MoS_2_, respectively, indicating the formation of the hexagonal phase of MoS_2_. The chemical structures of MoS_2_ nanosheets and MWCNTs were analyzed using FT-IR, as shown in Figure 3b. The peaks at 3460 cm^−1^ and 1617 cm^−1^ correspond to the stretching vibrations of O–H and bending vibrations of hydroxyl groups [36,37]. Additionally, the peaks at 1051 cm^−1^ and 589 cm^−1^ are attributed to the stretching vibrations of the Mo–O and Mo–S groups, respectively. The peak at 1124 cm^−1^ indicates asymmetric stretching vibrations of S=O groups, indicating the formation of MoS_2_. In Figure 3c, the absorption peaks at 1147 cm^−1^ and 1628 cm^−1^ can be ascribed to the C–OH and C=O stretching vibrations of the carboxylic groups, respectively.

### 3.2. The Alignment of MWCNTs in Liquid Epoxy

**Figure 4:**
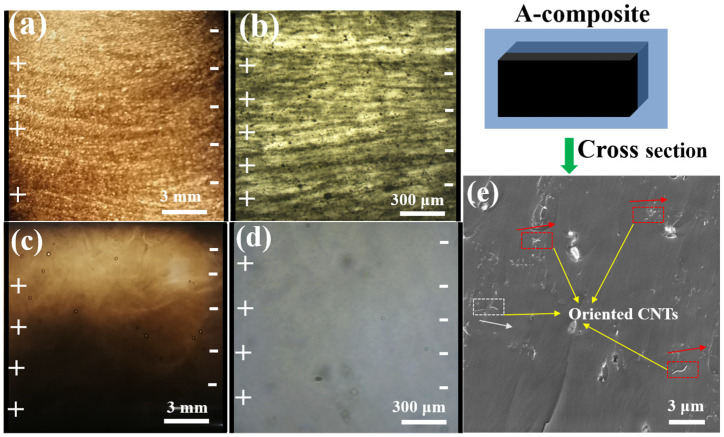
Digital image (**a**) and optical micrograph (**b**) of pristine MWCNTs alignment in liquid epoxy; Digital image (**c**) and optical micrograph (**d**) of oxidized MWCNTs alignment in the liquid epoxy; (All the epoxy suspension containing 0.05 wt% of MWCNTs + 0.05 wt% of MoS_2_); (**e**) SEM image of cross section of nanocomposite containing aligned 1.2 wt% of MWCNTs + 1 wt% of MoS_2_.

Figure 4 shows the MWCNTs alignment in the liquid epoxy and cured nanocomposite. Figure 4a,b clearly demonstrate the successful alignment of pristine MWCNTs in the liquid epoxy, forming aligned networks along the electric field direction while also revealing the presence of numerous agglomerates. In contrast, Figure 4c,d shows that it is difficult to observe aligned networks of MWCNTs in the epoxy suspension containing oxidized MWCNTs due to their excellent dispersion and alignment within the epoxy matrix [31]. In order to verify the form of MoS_2_ in the epoxy under DC electric field induction, the optical micrograph of liquid epoxy only containing untreated MoS_2_ nanosheets was investigated. It can be observed that the alignment of MoS_2_ did not occurred in liquid epoxy with DC electric field induction in Figure S1, further confirming that the aligned networks observed in liquid epoxy were indeed caused by the alignment of MWCNTs. The cross-section of the composite material in Figure 4c reveals the presence of aligned CNTs, indicating the formation of an aligned CNT structure within the cured epoxy nanocomposite. Moreover, the aligned CNTs can be observed on the surface of cross section of cured nanocomposite containing aligned 1.2 wt% of MWCNTs + 1 wt% of MoS_2_ from Figure 4e, also indicating the formation of orientation structures.

### 3.3. Characterization of Epoxy Nanocomposites

**Figure 5:**
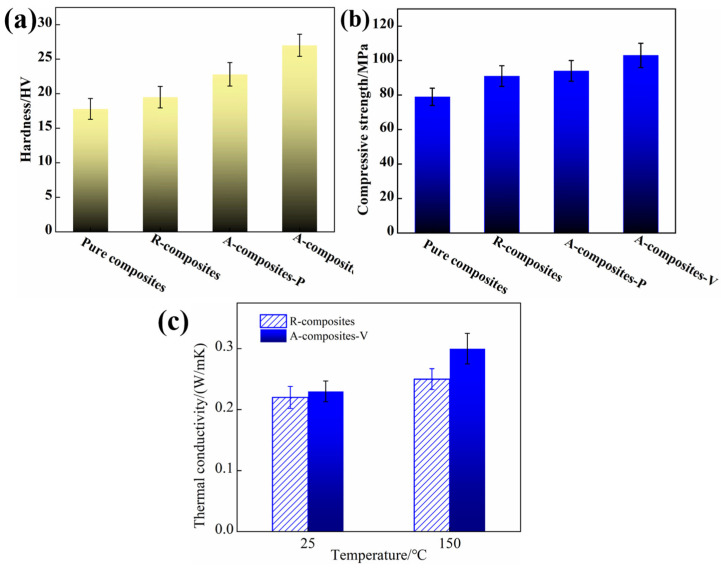
Vickers hardness (**a**), compressive strength (**b**) of epoxy nanocomposites, and thermal conductivity (**c**) of epoxy nanocomposites containing 1 wt% MoS_2_ and 1.2 wt% MWCNTs in different directions.

Figure 5 shows the mechanical properties and thermal conductivity of pure epoxy and epoxy nanocomposites, in addition to 1 wt% MoS_2_ and 1.2 wt% MWCNTs at different directions. Figure 5a,b indicate that the addition of nanoparticles to epoxy significantly enhances its hardness and compressive strength. Moreover, the hardness and compressive strength of epoxy nanocomposites containing aligned nanoparticles are higher compared to those with random nanoparticles. In particular, the hardness and compressive strength along the axial direction of MWCNTs are higher than those containing either randomly nanoparticles or the vertical to the axial direction of MWCNTs. This remarkable improvement can be attributed to the well-ordered MWCNTs in nanocomposites as well as their anisotropy.

The thermal conductivity of A-composites-V is superior to that of R-composites at room temperature, as shown in Figure 5c. As the temperature increases, both composites exhibit further enhancement in thermal conductivity, and A-composites-V demonstrates the most significant improvement, which could have a more important influence on the tribological properties of epoxy nanocomposites under a higher temperature.

### 3.4. Tribological Performances of Epoxy Nanocomposites

**Figure 6:**
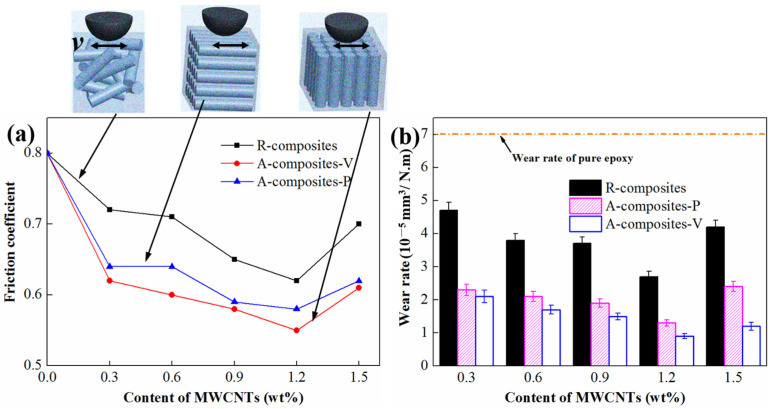
Friction coefficient (**a**) and wear rate (**b**) of epoxy nanocomposites containing 1 wt% MoS_2_ and different contents of MWCNTs under a load of 5 N.

Figure 6 shows the tribological performances of epoxy nanocomposites containing 1 wt% MoS_2_ and different contents of MWCNTs under various sliding conditions. It is evident that the presence of nanoparticles significantly reduces both the friction coefficient and wear rate of the epoxy nanocomposites compared to pure epoxy. In addition, the A-composites demonstrate anisotropic tribological properties, and the A-composites-V possess superior properties. Additionally, the A-composites-P shows better tribological properties compared to the R-composites for all MWCNTs content levels. When the contents of MWCNTs are below 1.2 wt%, the friction coefficient and wear rate decrease as the content of MWCNTs increases, then increase when the contents of MWCNTs increase to 1.5 wt%. The epoxy nanocomposites, therefore, achieved the lowest friction coefficient and wear rate with the addition of 1.2 wt% MWCNTs and 1 wt% MoS_2_. In this case, the friction coefficient of R-composites, A-composite-P, and A-composite-V decreased by 22.5, 27.5, and 31.3%, respectively, and the wear rate decreased by 61.4, 81.4, and 97.1%, respectively, compared to the pure epoxy under a load of 5 N.

**Figure 7:**
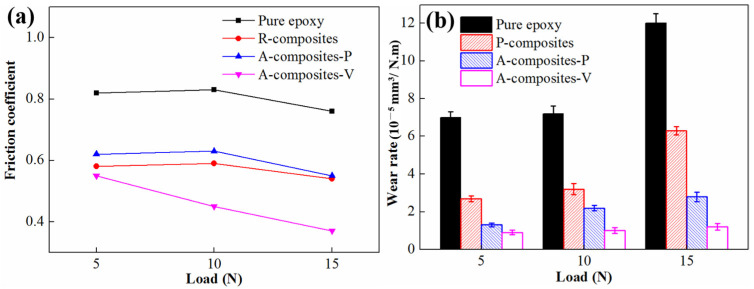
Friction coefficient (**a**) and wear rate (**b**) of epoxy nanocomposites containing 1 wt% MoS_2_ and 1.2 wt% MWCNTs under different loads.

It can be seen from Figure 7 that when the epoxy nanocomposites containing 1.2 wt% MWCNTs and 1 wt% MoS_2_, the friction coefficient of R-composites and A-composites-P increase slightly with a load ranging from 5 N to 10 N, then decrease with the load ulteriorly increased to 15 N. However, the wear rate increases as the load increases. The friction coefficient and wear rate of A-composites-V decrease with the load increase.

**Figure 8:**
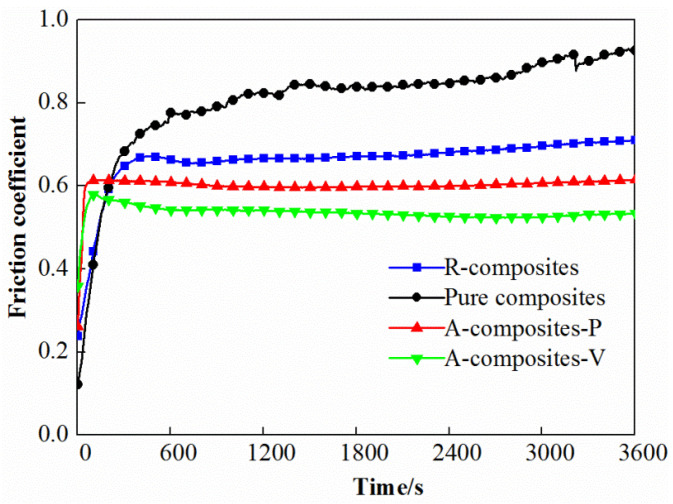
The friction coefficient vs. time of the different epoxy nanocomposites containing 1.2 wt% MWCNTs and 1 wt% MoS_2_ under a load of 5 N.

Figure 8 shows the friction coefficient of pure epoxy and its nanocomposites containing 1.2 wt% MWCNTs and 1 wt% MoS_2_ under a load of 5 N versus sliding time. The results indicate that the epoxy nanocomposites exhibit superior stability of the friction coefficient compared to pure epoxy while also exhibiting shorter running-in stages for A-composites when compared to R-composites and pure epoxy.

When the nanoparticles are at a lower content, the hardness of the epoxy nanocomposites will increase as the additives content increases and the higher hardness leads to better lubricating properties [38]. In addition, the number of fillers in the transfer films will increase, caused by increased concentration of fillers, resulting in a lower friction coefficient. Meanwhile, the increased hardness enhances the load-carrying capacity of nanocomposites [39], leading to higher wear resistance. As the MWCNTs content exceeds suitable content (1.2 wt%), the nanoparticles agglomerate could occur, resulting in a stress concentration around the particles and micro cracks. The nanoparticles thus tend to detach from the composite’s surface under the shear force, leading to abrasive wear and an increased wear rate.

The A-composites exhibit better tribological properties compared to R-composites, and the tribological properties of A-composites-V are better than A-composites-P. The reason can be attributed to the different alignment modes of nanoparticles. Furthermore, the content of nanoparticles near the surface of A-composites-V is higher than that of A-composites-P and R-composites due to the migration of the MWCNTs from cathode to anode under the DC electric field [30], thereby contributing to the enhancement of tribological performances of A-composites-V.

Temperature is also an important influence factor on the tribological properties of epoxy composites [1,40]. The temperature on the surface of epoxy nanocomposites increases when the load increases, leading to a reduction in hardness and subsequently resulting in a lower friction coefficient. Meanwhile, the real contact area of counterpart and epoxy nanocomposites increases at a higher load, resulting in the larger transfer films forming and, subsequently, a lower friction coefficient. The adhesive wear is more likely to occur under higher temperatures, resulting in a significant increase in wear rate as the load increases. However, due to its higher thermal conductivity, A-composites-V exhibits a relatively lower surface temperature compared to other epoxy nanocomposites. Consequently, the hardness of A-composites-V is relatively higher, resulting in enhanced wear resistance [23,41]. The adhesive wear is less prone to occur for A-composites-V under relatively lower temperatures, and hence, the wear rates of A-composites-V increase slightly with the load increased.

### 3.5. Analysis of Worn Surface

**Figure 9:**
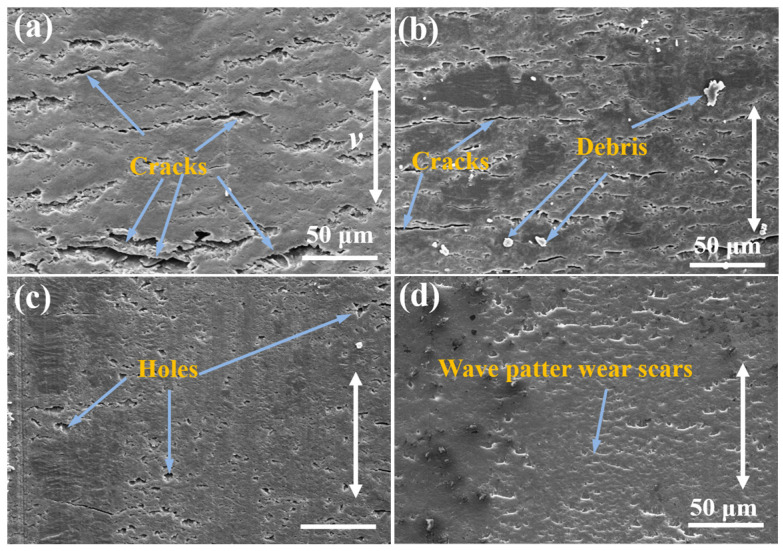
SEM micrographs of worn surfaces under a load of 5 N: (**a**) pure epoxy, (**b**) R-composites containing 1.2 wt% MWCNTs and 1 wt% MoS_2_, (**c**) A-composites-P containing 1.2 wt% MWCNTs and 1 wt% MoS_2,_ and (**d**) A-composites-V containing 1.2 wt% MWCNTs and 1 wt% MoS_2_.

**Figure 10:**
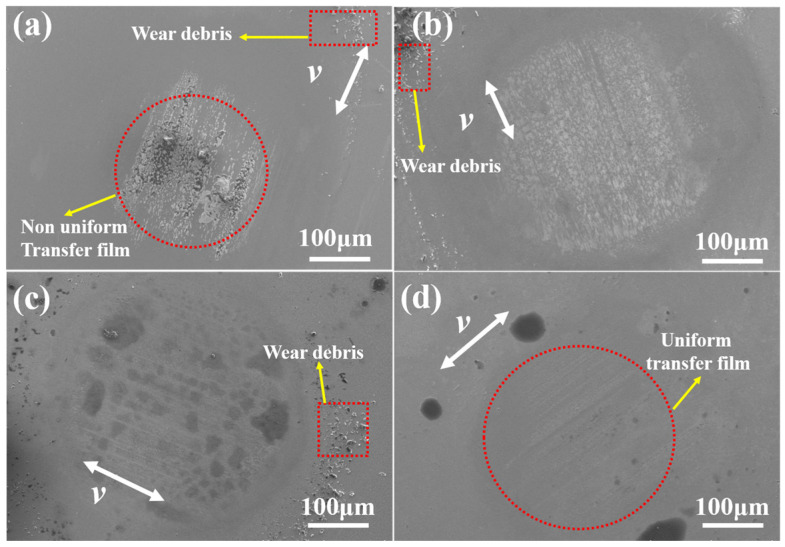
SEM micrographs of worn surfaces of counterpart against (**a**) pure epoxy, (**b**) R-composites containing 1.2 wt% MWCNTs and 1 wt% MoS_2_, (**c**) A-composites-P containing 1.2 wt% MWCNTs and 1 wt% MoS_2_, and (**d**) A-composites-V containing 1.2 wt% MWCNTs and 1 wt% MoS_2_ under a load of 5 N.

The worn surface of epoxy nanocomposites and corresponding counterparts are shown in Figure 9 and Figure 10. The number of cracks can be observed on the surface of pure epoxy in Figure 9a, primarily caused by plastic deformation. Correspondingly, Figure 10a reveals a thick transfer film and some debris on its surface. These results indicate that the wear mechanism shows characteristics of severe adhesive and fatigue wear. For R-composites, some small debris and cracks on their surface can be seen in Figure 9b. Meanwhile, the relatively thinner transfer film and more debris can be observed on its counterpart surface, indicating that fatigue and abrasive wear may occur during the sliding process because of the higher load carrying and hardness of nanocomposites.

In comparison to Figure 9a,b, the surface of A-composites-P in Figure 9c exhibits minimal cracks instead of a few holes. Moreover, the surface appears relatively flat, while the surface of the counterpart is relatively smooth, as seen in Figure 10c, indicating that plastic deformation and fatigue wear are less prone to occur. The reason can be attributed to the stronger mechanical interlocking of MWCNTs with the matrix along the axial direction of MWCNTs, where slight abrasive wear is the main wear mode. The absence of cracks and holes in Figure 9d indicates that no observable wear scars were generated on the surface of A-composites-V. The obvious debris is not observed on the surface of the counterpart surface from Figure 10d and is covered with a thin transfer film. As a result, the A-composites-V exhibits the best tribological properties.

**Figure 11:**
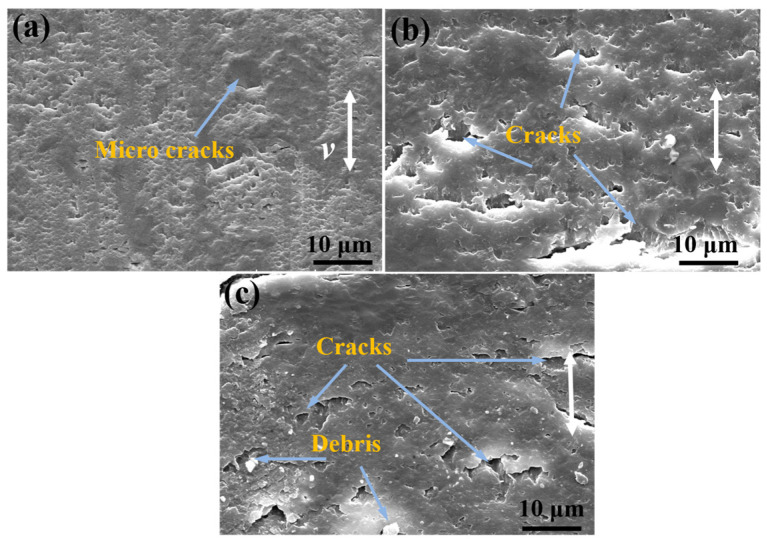
SEM micrographs of worn surfaces of A-composites-V containing 1.2 wt% MWCNTs and 1 wt% MoS_2_ under different loads of (**a**) 5 N, (**b**) 10 N, and (**c**) 15 N.

Figure 11 shows the worn surface of A-composites-V containing 1.2 wt% MWCNTs and 1 wt% MoS_2_ under different loads. The presence of micro cracks on the surface of A-composites-V under a 5 N load can be clearly observed in Figure 11a. As the load increases to 10 N, surface cracks are generated (Figure 11b). With the load increasing to 15 N, it can be seen that, other than the cracks and some debris observed on the surface of A-composites-V (Figure 11c), the surface is relatively smoother than that of sliding under a load of 10 N. The reason for this can be ascribed to three aspects. The anisotropic structure of MWCNTs contributes to the improvement of tribological and mechanical properties of epoxy nanocomposites after alignment treatment [42].

**Figure 12:**
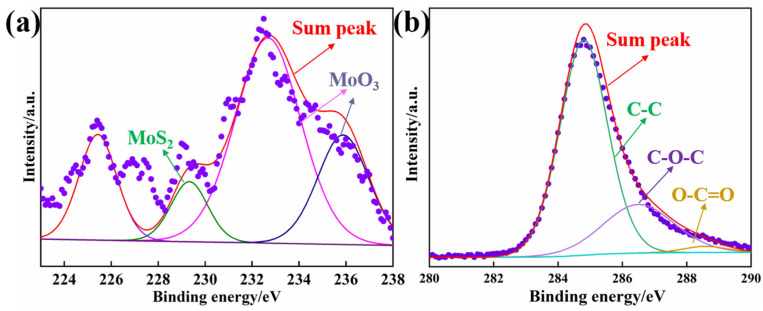
XPS profiles of worn surface of A-composites-V containing 1.2 wt% MWCNTs and 1 wt% MoS_2_ under load of 5 N: (**a**) Mo3d, (**b**) C1s.

The XPS profiles of the wear surface of A-composites-V containing 1.2 wt% MWCNTs and 1 wt% MoS_2_ under a load of 5 N are presented in Figure 12. In Figure 12a, the main peak located at 229.45 eV belongs to a characteristic peak of MoS2, while the peaks at 232.67 and 235.88 eV indicate the presence of MoO_3_, suggesting the formation of a shear layered structure of MoS_2_, whereas oxidation occurs leading to MoO_3_ formation during frictional processes. From Figure 2, C1s exhibits distinct characteristic peaks of C-C, C-O-C, and O-C=O bonds, located at peaks of 285.32, 286.65, and 288.55 eV, respectively. This indicates damage to the MWCNTs and the formation of carbon film on the surface of the composite due to friction and shear forces, which contribute to lubrication and anti-wear properties.

### 3.6. Wear Mechanism of MWCNTs Direction for Epoxy Nanocomposites

**Figure 13:**
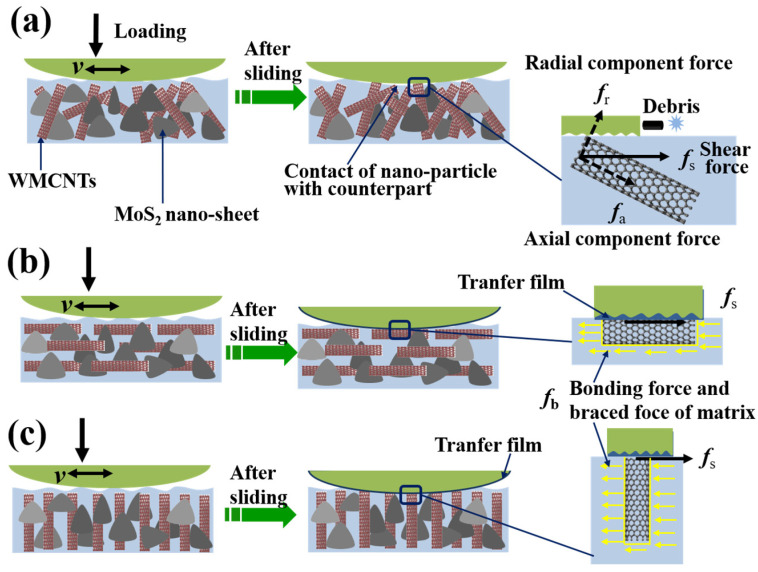
Wear mechanism of epoxy nanocomposites with various MWCNTs direction: (**a**) sliding along the random direction of MWCNTs, (**b**) sliding along the axial direction of MWCNTs, (**c**) sliding along the radial direction of MWCNTs.

The tribological mechanism of epoxy nanocomposites containing aligned MWCNTs is proposed in Figure 13. When the counterpart contacts with MWCNTs at an angle with epoxy nanocomposites containing random alignment MWCNTs, the MWCNTs will be subject to a shear force along the sliding direction. The shear force will decompose into radial component force and axial component force. The MWCNTs thus tend to detach from epoxy nanocomposite surfaces, resulting in the production of debris (Figure 13a). By contrast, when the axial direction of MWCNTs is parallel or vertical to the sliding direction, the MWCNTs tend to detach less from the epoxy nanocomposites’ surface due to the horizontal shear force, normal pressures, as well as interface bonding and braced force of matrix. The related enhancing mechanism is shown in Figure 13b,c. As a result, the A-composites possess higher wear resistance compared to the R-composites. Moreover, the MWCNTs have a smaller stress surface and larger normal depth when their axial direction is perpendicular to the sliding direction compared to when the sliding direction is parallel to its axial direction. The MWCNTs are thus hard to detach from epoxy nanocomposite surfaces, the A-composites, therefore, obtain excellent tribological properties in the vertical direction.

## 4. Conclusions

The epoxy nanocomposites containing MoS_2_ nanosheets and aligned MWCNTs have been fabricated by using DC electric field inducement. The mechanical and tribological properties of epoxy nanocomposites can be significantly improved by the addition of 1.2 wt% MWCNTs and 1 wt% MoS_2_ nanosheets compared to pure epoxy. Furthermore, when the MWCNTs are aligned in nanocomposites, a maximum decrease of 11.3 and 66.7% in friction coefficient and wear rate were achieved compared to R-composites when the sliding direction was perpendicular to the axial direction of MWCNTs under a load of 5 N. Meanwhile, the hardness and compressive strength increased by 51.7% and 30.4% along the axial direction of MWCNTs compared to pure epoxy, respectively. Furthermore, the friction coefficient of A-composites-V containing 1.2 wt% MWCNTs and 1 wt% MoS_2_ nanosheets decreased when the load increased and reached 0.37 under a load of 15 N. The wear rate increased with the load increase and reached 0.9 × 10^−5^ mm^3^/N.m under a load of 5 N. The increased temperature conductivity and mechanical properties caused by aligned MWCNTs enhance the tribological properties of epoxy nanocomposites. In addition, the unique alignment mode of MWCNTs and the higher surface content of nanoparticles caused by electric field inducement are also considered to be the reasons for the improved tribological properties.

## Data Availability

The original contributions presented in the study are included in the article/Supplementary Material, further inquiries can be directed to the corresponding author.

## Supplementary Materials

The following supporting information can be downloaded at https://www.mdpi.com/article/10.3390/ma17194745/s1, Figure S1: Optical micrographs of liquid epoxy containing 0.1 wt% MoS_2_ nanosheets (a) before and (b) after DC electric field induction. References [29,43] are cited in the Supplementary Material.
