# Supplementary material for: Fabrication and Tribological Properties of Epoxy Nanocomposites Reinforced by MoS2 Nanosheets and Aligned MWCNTs
Source: Materials (Basel). 2024 Sep 27;17(19):4745. doi: 10.3390/ma17194745 (PMC11478268; doi:10.3390/ma17194745)
Supplement: Supplementary file 1 [file materials-17-04745-s001.zip › materials-3160024-supplementary.pdf]

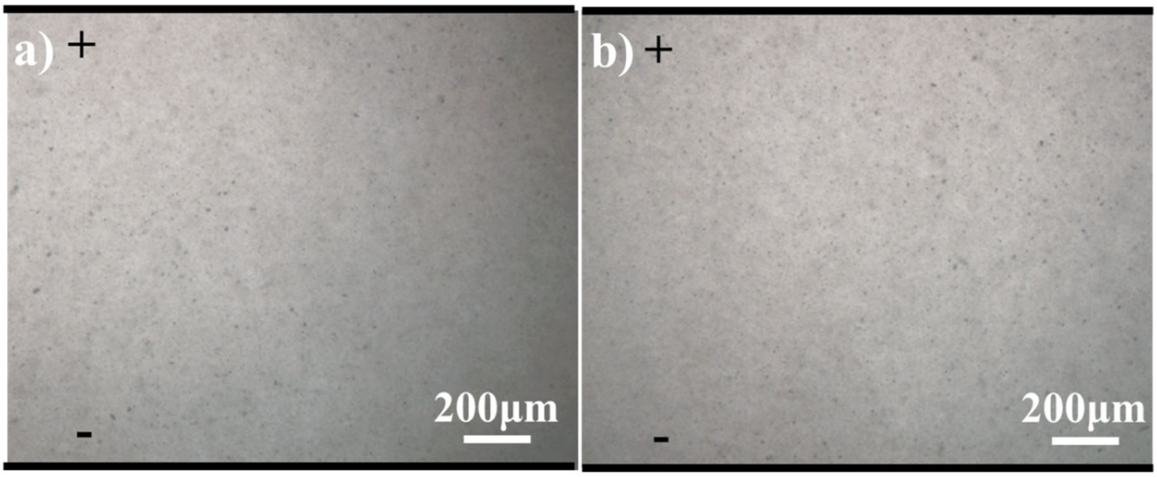

Figure S1. Optical micrographs of liquid epoxy containing 0.1 wt% MoS_2 nanosheets (a) before and (b) after DC electric field induction.

In order to verify the form of MoS_2 nanosheets in the epoxy under DC electric field induction, the optical micrograph of liquid epoxy only containing untreated 0.2 wt% MoS_2 after DC electric field (70 $V_{p\text{-}p}$/mm, 5 kHz) induction for 90 min was studied. The untreated MoS_2 nanosheets were used for ease of observation of the formation of MoS_2 nanosheets in liquid epoxy, and the alignment time was set according to the curing time of epoxy.

It is clear that the alignment of MoS_2 nanosheets in the epoxy did not occur under DC electric field induction, and the epoxy suspension was uniform and stable in Figure S1. Generally, conductive or dielectric particles can achieve alignment in a dielectric liquid with electric field induction [1]. The alignment time of fillers can be calculated according to the Equation (S1) [2]:

$$t = \frac{2x_0^3}{3B} \qquad \text{(S1)}$$

where

$$B = \frac{4\pi a^4}{9\eta k_t \varepsilon_0} \frac{E^2 \varepsilon_m^2}{\left(\frac{\pi}{2} \frac{b}{a}\right)^2} \qquad \text{(S2)}$$

η is the liquid epoxy viscosity, ε_0 is the vacuum permittivity, and E is the electric field strength. k_r is the rotational friction coefficient and $k_r = 32\ a^3/3$, ε_m is the permittivity of the matrix, and x_0 is the initial distance between the closest opposite charged ends

of the MoS_2 nanosheet, which depends on the content of the nanosheet in the liquid epoxy, and can be given by Equation (S3):

$$x_0 = \frac{1}{\rho}\frac{m_s}{W_s}\frac{1}{4a^2} \quad \text{(S3)}$$

where ρ is the density of the epoxy, W_s is the weight fraction of MoS_2 nanosheets in epoxy, m_s is the mass of each MoS_2 nanosheet, and a and b are semi-major and semi-minor axes of MoS_2 nanosheet. In this study,

$\eta = 1.1$ Pa.s, $\varepsilon_0 = 8.85 \times 10^{-12}$ F/m, $\varepsilon_m = 3.5$, E = 70000 V/m, $a = 3 \times 10^{-7}$ m, $b = 1.5 \times 10^{-7}$ m, $\rho = 1.16 \times 10^3$ kg/m^3, $W_s = 0.2$ wt%.

The time of alignment can be estimated as $t \approx 1.93 \times 10^{20} \times m_s^3$. In this study, the aligned MoS_2 nanosheets were not observed, and the reason can be ascribed to the larger mass of a single MoS_2 nanosheet, which takes a long time to achieve the alignment. The as-prepared MoS_2 nanosheets may be not suitable dielectric particles in this epoxy system, resulting in the alignment not being able to occur.

References:

[1] L. R. Holmes, J. C. Riddick, Research Summary of an Additive Manufacturing Technology for the Fabrication of 3D Composites with Tailored Internal Structure. Jom-Us. 66 (2014) 270-274.

[2] S. Wu, R. B. Ladani, J. Zhang, E. Bafekrpour, K. Ghorbani, A. P. Mouritz,et al.C. H. Wang, Aligning multilayer graphene flakes with an external electric field to improve multifunctional properties of epoxy nanocomposites. Carbon. 94 (2015) 607-618.
